# Effect of Exercise on Metabolic Syndrome Variables in Breast Cancer Survivors

**DOI:** 10.1155/2013/168797

**Published:** 2013-11-11

**Authors:** Gwendolyn A. Thomas, Marty Alvarez-Reeves, Lingeng Lu, Herbert Yu, Melinda L. Irwin

**Affiliations:** ^1^Yale University, New Haven, CT, USA; ^2^Dana Farber Cancer Institute, Boston, MA, USA; ^3^Yale School of Public Health, New Haven, CT, USA; ^4^University of Hawaii Cancer Center, Honolulu, HI, USA

## Abstract

*Objective*. Breast cancer survivors are highly sedentary, overweight, or obese, which puts them at increased risk for comorbid chronic disease. We examined the prevalence of, and changes in, metabolic syndrome following 6 months of an aerobic exercise versus usual care intervention in a sample of sedentary postmenopausal breast cancer survivors. *Design and Methods*. 65 participants were randomized to an aerobic exercise intervention (EX) (*n* = 35) mean BMI 30.8 (±5.9) kg/m^2^ or usual care (UC) (*n* = 30) mean BMI 29.4 (±7.4) kg/m^2^. Metabolic syndrome prevalence was determined, as well as change in criteria and overall metabolic syndrome. *Results*. At baseline, 55.4% of total women met the criteria for metabolic syndrome. There was no statistically significant change in metabolic syndrome when comparing EX and UC. However, adhering to the exercise intervention (at least 120 mins/week of exercise) resulted in a significant (*P* = .009) decrease in metabolic syndrome *z*-score from baseline to 6 months (−0.76 ± 0.36) when compared to those who did not adhere (0.80 ± 0.42). *Conclusions*. Due to a higher prevalence of metabolic syndrome in breast cancer survivors, lifestyle interventions are needed to prevent chronic diseases associated with obesity. Increasing exercise adherence is a necessary target for further research in obese breast cancer survivors.

## 1. Introduction

Obesity and sedentary lifestyle are associated with a higher risk of breast cancer recurrence and onset of comorbid conditions in women diagnosed with breast cancer [1–4]. Metabolic syndrome is a clustering of symptoms that includes abdominal adiposity, hypertension, dyslipidemia, and glucose dysregulation which can markedly increase the risk of insulin resistance, diabetes, stroke, and cardiovascular disease. Prior research has demonstrated that between 50% and 64% of the 2.5 million breast cancer survivors are either overweight (BMI 25–30 kg/m^2^) or obese (BMI > 30 kg/m^2^) [5–7]. Additionally, breast cancer survivors are more likely to spend greater than 8 hours a day in a sedentary state when compared to noncancer participants [8]. Breast cancer has been associated in several studies with metabolic syndrome [2, 5] and insulin resistance [2, 4, 6–9]. Given that metabolic dysregulation may affect risk for recurrence of breast cancer and onset of additional chronic disease [10], investigation into effective interventions for reducing metabolic syndrome in breast cancer survivors is a much needed area of research. Physical activity may be an effective intervention for preventing and/or improving metabolic syndrome variables, thereby reducing risk for additional associated chronic diseases. 

Recent research has shown that metabolic syndrome variables can be improved by lifestyle modification in the general population. Prior investigations have either prescribed a dietary intervention that reduced dietary fat intake and promoted weight loss [11], prescribed exercise alone with no control condition [12], or prescribed a combined weight loss and exercise intervention [13]. In a study in healthy postmenopausal obese women, investigators found that a walking program, three days a week for 16 weeks, reduced individual criteria, but not overall prevalence of metabolic syndrome in individuals who had two or more criteria of metabolic syndrome [14]. Additionally, aerobic exercise protocols that resulted in a concomitant reduction in weight over an 18-month period have shown reductions in metabolic syndrome criteria [15]. One prior study examined exercise, diet, and a combination of the two compared to control as an intervention for metabolic syndrome in men and postmenopausal women with dyslipidemia [16]. They found that associations between exercise and diet with metabolic syndrome variables were accounted for by body fat loss. Given the observed benefits of physical activity interventions on metabolic syndrome variables in other clinical populations, it is important to understand whether these effects generalize to breast cancer survivors, a population where having the metabolic syndrome may increase their risk for recurrence [10]. It is also important to examine whether change in body weight or body fat accounts for any observed improvements in metabolic syndrome as this may provide insight into the mechanisms linking exercise to the metabolic syndrome. However, there are few, if any, randomized control trials that have been published examining the effects of exercise on changes in the metabolic syndrome in women diagnosed with breast cancer.

Given the limited prior literature, the purpose of this study was to investigate the prevalence of the metabolic syndrome at baseline and to examine the effect of aerobic exercise versus usual care over 6 months on improving metabolic syndrome criteria and overall metabolic syndrome score in sedentary postmenopausal breast cancer survivors. 

## 2. Methods and Procedures

### 2.1. Participants

Participants were 65 postmenopausal breast cancer survivors who were enrolled in the Yale Exercise and Survivorship (YES) study that has been described in detail elsewhere [17]. Participants were within 1–10 years of diagnosis of stages 0–111A breast cancer and had completed chemotherapy and/or radiation at least 6 months before enrollment. Inclusion criteria required participating in less than 90 minutes of physical activity per week prior to enrollment; participants were nonsmokers and were free of other serious health problems. Only those women who were sedentary or reported less than 90 minutes of moderate to vigorous physical activity per week and were not currently participating in a weight loss diet program were eligible. Exclusion criteria for the study included women younger than 40 years of age due to potential differences in disease etiology and women over 75 years of age due to likelihood of significant comorbidities and safety concerns for elderly exercise participants.

### 2.2. Recruitment

We used the Yale-New Haven Hospital Tumor Registry to obtain the names of Connecticut women diagnosed with breast cancer by any Yale-affiliated physician from March 2004 to January 2006. Staff contacted each patient's physician to request permission to contact the participant. An invitation letter was mailed to the participant, followed by a telephone screening questionnaire. From 788 screening calls made, 75 (9.5%) women were eligible, interested, and randomized. Fasting blood was available for 65 of the women.

### 2.3. Anthropometric, Blood Pressure, and Dual Energy X-Ray Absorptiometry (DXA) Measurement

At baseline and six months, measurements of weight, height, waist circumference, and blood pressure were taken twice in succession by the same technician and averaged for analysis. Weight was measured on an electronic scale and recorded to the nearest 0.1 kg and height was measured with a standard stadiometer, rounding up to the nearest 0.5 cm. One blood pressure measurement was taken at rest with the participant sitting. Dual-energy X-ray absorptiometry (DXA) (Hologic 4500 with a “Discovery” upgrade, Hologic Inc., Waltham, MA, USA) scans were performed to assess total body fat and lean mass. 

### 2.4. Physical Activity Measurement

At baseline and 6 months, participants completed an interview-administered physical activity questionnaire, which was used to assess the past 6 months of recreational activity [17] and a seven-day physical activity log [18]. Women recorded the type and duration of any physical activity done on each day for the physical activity log. Additionally, hours per week of moderate to vigorous intensity aerobic activity were determined using Ainsworth Compendium of Physical Activities [19].

### 2.5. Medical History and Medications

An interviewer-administered questionnaire was also administered at baseline to collect relevant medical history as well as current medication usage, health habits, and comorbidities. The questionnaire was designed to collect information about history and/or treatment of medical conditions, such as heart disease, high blood pressure, arthritis, diabetes, and cancer, as well as medical symptoms over the past 30 days, and prior and concomitant medications. Additional information on disease stage, hormone-receptor status, histological grade, therapy and evidence of completion, and surgery was provided by participants at baseline and 6 months. The information from these questionnaires was later confirmed by the participant's physician and the review of medical records.

### 2.6. Food Frequency Questionnaire

All participants completed a 120-item food frequency questionnaire at baseline and 6 months [20]. Participants were told to maintain dietary intake as unchanged throughout the trial. 

### 2.7. Exercise Intervention

The participants in the exercise intervention were instructed to complete 150 minutes of moderate intensity aerobic activity which consisted of three weekly certified exercise trainer supervised exercise sessions at a local health club and two weekly unsupervised exercise sessions. Exercise sessions consisted primarily of walking, which is a preferred activity for breast cancer survivors. However, participants could meet the exercise goal through other forms of aerobic activity such as stationary biking and elliptical training. Resistance exercise and yoga were excluded activities and did not count towards the exercise goal for each week as they did not involve sustained aerobic effort. Participants completed three 15-minute sessions during Week 1 and gradually built up to five 30-minute moderate intensity sessions by Week 5 which is consistent with the American College of Sports Medicine (ACSM) exercise guidelines for adults. Exercise started at 50% of predicted maximal heart rate (220-age) and was gradually increased in accordance with approximately 60–80% of predicted maximal heart rate. Participants wore heart rate monitors for each exercise session to enable self-monitoring of exercise intensity (Polar Electro, Woodbury, NY). Following each exercise session, participants recorded the type, duration, perceived intensity of activity, and average heart rate during exercise in physical activity logs, which were repeated on a weekly basis. The physical activity logs ensured weekly compliance and were used to determine exercise intensity for the following week. 

### 2.8. Usual Care Group

Women in the usual care group were instructed to continue with their usual activities. If a participant wanted to exercise, she was told she could, but the exercise program and training materials would not be offered to her until the end of the study. At the end of the trial, women in the usual care condition were offered three supervised training sessions, a pedometer, exercise handouts, and the results of their clinical tests. Additionally, all study participants received quarterly newsletters that highlighted issues relevant to breast cancer survivorship.

### 2.9. Blood Draw and Metabolic Variable Assays

Fasting blood draws were collected at the baseline and 6-month visit and plasma samples were stored at −80°C until assayed. Plasma total cholesterol (TC), high-density lipoprotein (HDL), triglycerides, and glucose were enzymatically measured on an Alfa Wassermann ACE Alera Chemistry Analyzer (Alfa Wassermann, West Caldwell, NJ, USA) with reagents supplied by the company. Intra assay coefficients of variation were as follows: TC 1.1% HDL 2.0%, triglycerides 1.2%, and glucose 0.9%. Interassay coefficents of variation were as follows: TC 1.6%, HDL 4.3%, triglycerides 1.8%, and glucose 1.7%. 

### 2.10. Metabolic Syndrome Criteria

Based on the US National Cholesterol Education Program Adult Treatment Panel III (ATPIII) definition [21], metabolic syndrome was defined as the presence of ≥3 of the following risk factors: waist circumference (WC) ≥ 88 cm, triglycerides (TG) ≥ 150 mg/dL, or taking medication to lower cholesterol; HDL cholesterol < 50; systolic blood pressure (SysBP) ≥ 130 mm Hg or ≥85 mm Hg diastolic blood pressure (DiasBP) or taking blood pressure medication; and fasting glucose ≥ 100 mg/dL or taking diabetes medication.

### 2.11. Metabolic Syndrome *z*-Score

Consistent with prior publications examining a dimensional score for metabolic syndrome [22–24], we created a modified *z*-score for each metabolic syndrome variable and summed for a total score ((50-HDL)/14.5) + ((TG-15)/52.4) + ((Glucose–100)/11.75) + ((WC–88)/13.75) + ((SysBP-130)/15.7) + ((DiasBP-85)/7.9). 

### 2.12. Statistical Analyses

Metabolic syndrome onsets and offsets were coded and tested for significant differences using a likelihood ratio chi-square statistic. A general linear model (GLM) controlling for baseline scores and age was implemented in SAS to examine effects over time between the intervention and usual care groups on the metabolic syndrome. Statistical significance was assumed for *P* ≤ 0.05. Exercisers were further classified as adherers if they participated in greater than or equal to 80% of the recommended amount (which is commonly defined as adherent) of 150 min/wk (i.e., 120 minutes of exercise week (*n* = 20)) or nonadherers if they participated in less than 120 minutes per week (*n* = 15). Changes in metabolic syndrome variables and overall score were examined between adherers and nonadherers, controlling for baseline values and age.

## 3. Results

Demographic and clinical characteristics at baseline in women randomized to exercise versus usual care are shown in Table 1. There were no significant differences between groups at baseline. The percentage of participants at baseline who had metabolic syndrome was 55.4% (see Table 2). At baseline, 24 of the 35 (69%) women randomized to exercise and 12 of 30 (40%) women randomized to usual care met criteria for metabolic syndrome. At 6-month followup, 20 of 35 women in the exercise group (57%) had metabolic syndrome, whereas 13 of 30 women (43%) in the usual care group had metabolic syndrome. At baseline, the average number of physical activity minutes per week was 13.0 minutes per week for the exercise group and 12.0 minutes per week for the usual care group. The frequency distribution of number of metabolic syndrome criteria is also displayed in Table 2. Results from the Chi-square test indicate a significant difference in metabolic syndrome onsets between groups with more onsets in the usual care group at 6 months (*χ* ^2^ = 6.49, *P* = 0.01).

At 6 months, the exercise group had a significant increase in moderate to vigorous intensity recreational activity compared to the usual care group (129 minutes/week versus 45 minutes/week, *P* < 0.001). The exercise goal was 150 min/wk of moderate intensity aerobic exercise; 33% of women achieved this amount. 57% of women achieved 80% of the exercise goal or 120 min/wk, and 75% of women achieved 90 min/wk.

Table 3 shows the mean baseline and six-month metabolic syndrome change scores by intervention group for metabolic syndrome criteria. There was no statistically significant difference in the baseline to 6-month metabolic syndrome *z*-score between exercisers and controls; however, fasting blood glucose significantly decreased in the exercise group compared with a slight increase among women in the usual care group (−1.3 ± 1.2 versus 0.6 ± 1.3, *P* < 0.029).

Adjusting or stratifying by baseline to 6-month change in body weight or body fat did not change the overall main effects. Exercise adherers continued to have significant improvement in metabolic syndrome *z*-score even when change in body fat mass was controlled for (−0.69 ± 0.37 adherers versus 0.70 ± 0.43 nonadherers; *P* = .024). Exercise adherers also had significant improvements in metabolic syndrome *z*-score when change in lean mass was controlled for (−0.73 ± 0.36 adherers versus 0.76 ± 0.42 nonadherers; *P* = .012). 

Table 4 shows change in metabolic syndrome *z*-score and criteria stratified by exercise adherence. Exercise adherers increased HDL cholesterol relative to nonadherers (2.91 ± 1.42 versus −2.67 ± 1.64, *P* < .016). In addition, a decrease in Metabolic Syndrome *z*-score was greater for the exercise adherers than for the nonadherers (−0.76 ± 0.37 versus 0.80 ± 0.42, *P* < .009). Exercise adherers continued to have significant improvement in metabolic syndrome *z*-score even when change in body fat mass was controlled for (−0.69 ± 0.37 adherers versus 0.70 ± 0.43 nonadherers; *P* = .024). Exercise adherers also had significant improvements in metabolic syndrome *z*-score when change in lean mass was controlled for (−0.73 ± 0.36 adherers versus 0.76 ± 0.42 nonadherers; *P* = .012).

## 4. Discussion

In our study, we observed that over half of our sample of breast cancer survivors were defined as having the metabolic syndrome, putting them at higher risk for other chronic diseases including cardiovascular disease and breast cancer recurrence [2, 25]. In addition, women randomized to the usual care group had higher rates of new onset of metabolic syndrome over the six months. A moderate intensity aerobic exercise program was not associated with an improvement in a metabolic syndrome *z*-score over six months; however, exercise was associated with a statistically significant decrease in fasting glucose after 6 months. The amount of exercise performed is also of importance, as we observed a decrease in the metabolic syndrome *z*-score and an increase in HDL cholesterol in women participating in at least 80% of the recommended amount of physical activity (i.e., 120 min/wk) compared with women participating in less than 120 min/wk of exercise. The prevalence rate of metabolic syndrome in this sample (55.4%) of breast cancer survivors who were evaluated at least 6 months after cancer treatment replicates and extends [26] prior findings from Porto and colleagues who found a 59.2% prevalence rate of metabolic syndrome in newly diagnosed breast cancer patients. This finding is particularly salient when compared to the prevalence rate of 37% in age and gender matched individuals in the general population [27]. This also suggests that prevalence rates of metabolic syndrome in breast cancer patients remain similar before and after cancer treatment. Individual components of metabolic syndrome such as higher blood pressure, dyslipidemia, and abdominal obesity are closely related to the etiology and prognosis of breast cancer. Only a few studies have investigated the prevalence of metabolic syndrome in breast cancer survivors and to our knowledge, none have examined the effect of an aerobic exercise intervention on metabolic syndrome criteria and overall metabolic risk score. Further longitudinal investigations are needed to determine whether metabolic syndrome rates increase during the course of treatment and whether exercise prescription may play an important role in decreasing metabolic syndrome risk for this population. 

At baseline, both the exercise group and the usual care group had extremely low levels of physical activity (average of 13 and 12 minutes per week, resp.) and 68% of the participants reported no weekly physical activity. Given that 50% of breast cancer survivors are obese [5] and predominantly have low levels of physical activity, this population is further put at risk for cancer recurrence and other comorbid chronic conditions. During the intervention, 20 of the 35 participants met 80% or 120 minutes of exercise per week, which reflects a clinically significant increase in physical activity from baseline. Although the dose of exercise in this intervention is modest, it signifies an obtainable goal for a population that is not likely to participate in physical activity. 

Although the exercise intervention did not yield overall changes in metabolic syndrome *z*-score, it did result in a reduction in fasting blood glucose, which replicates previous beneficial effects of exercise interventions in type II diabetes [28–30]. In other populations (of healthy men and women), exercise has shown the ability to reduce metabolic syndrome criteria. For example, exercise has decreased blood pressure [31], triglycerides [32] fasting blood glucose [28], and increased HDL cholesterol [27, 28] and improved metabolic risk score [23, 24]. Thus, it is surprising that the exercise intervention did not result in changes beyond those in glucose. However, dose and type of exercise may play an important role in understanding this discrepancy. Our findings that meeting 80% of the ACSM weekly exercise guidelines improves HDL and overall metabolic syndrome *z*-score in postmenopausal breast cancer survivors support the interpretation that dose of exercise is a vital component in reducing metabolic syndrome risk. This finding highlights that exercise, even at very modest amounts, can aid individuals in reducing chronic disease risk. Giving individuals who are not prone to high levels of physical activity an exercise prescription that is obtainable and feasible is an important tool in preventing cancer recurrence and comorbid chronic disease. Given the complexity of comorbid illness in breast cancer survivors and the high prevalence rates of metabolic syndrome in this population, further examination of the dose required to provide beneficial reductions in metabolic syndrome, and the mechanisms by which exercise conveys this benefit, is much needed. Although previous research found that changes in metabolic syndrome resulting from diet and exercise interventions were accounted for by change in fat mass [12, 29], our results suggest that changes in fat mass did not account for the beneficial effects of exercise on metabolic syndrome. 

This study demonstrates that sustained aerobic exercise provides health benefits that are relevant to metabolic health in breast cancer survivors. It is important to note that these health benefits are seen by exercising less than the ACSM recommended 220 minutes of exercise per week. This is in line with recent research which has shown a threshold in which further increase of exercise does not necessarily produce additional benefits to outcomes [33, 34]. This suggests a dose-dependent attenuation of these benefits. It is currently unclear what the optimal dose is, as it seems to be related to type of exercise performed, gender of participant, and other individual differences that research has not yet identified [34–38]. The moderate intensity aerobic exercise and the dose performed were well tolerated in these breast cancer survivors. The effects of physical activity on prognosis among breast cancer survivors have been examined by numerous randomized controlled trials and exercise has been deemed safe and a key factor in improving outcomes for cancer survivors [39]. Further research is now needed on the multitude of benefits from exercise to health status in breast cancer survivors and the optimal dose and type of exercise at which benefits are observed. 

A major strength of this study is the use of a continuous score for the metabolic syndrome which replicates previous studies in noncancer survivor samples [18, 19]. The metabolic syndrome *z*-score allows equal weighting of each risk factor on a dimensional scale and thus is more sensitive to overall change in metabolic syndrome criteria. Additional strengths include the randomized study design and use of a sedentary group of participants, which makes the results generalizable to the clinical population, and supervised exercise to ensure compliance with the study protocol. A limitation in this study is the small sample size which may limit statistical power with regard to some of our stratified analyses. An additional limitation of the present study was that the participants were exercising three times per week in a supervised setting and, thus, these results may not generalize to nonsupervised individuals.

## 5. Conclusions

In predominantly overweight or obese, physically inactive, breast cancer survivors, adherence to a moderate intensity aerobic exercise intervention was associated with improvements in metabolic syndrome criteria. Given the high prevalence rates observed in this sample, and prior samples, lifestyle interventions are needed to address the ongoing chronic disease issues associated with metabolic dysregulation in breast cancer survivors. Additional randomized control trials with larger sample sizes should examine the dosage and types of exercise that are most beneficial for metabolic syndrome improvements. 

## Figures and Tables

**Table 1:** Baseline characteristics of participants.

Characteristic mean (SD) or %	Exercise (*n* = 35)	Usual care (*n* = 30)	Nonadherers (*n* = 15)	Adherers (*n* = 20)
Age (yr)	56.5 (9.8)	55.1 (7.6)	57.5 (12.8)	55.7 (6.9)
Weight (kg)	82.1 (16.5)	77.2 (20.4)	86.6 (17.0)	78.7 (15.6)
Height (cm)	161.8 (6.2)	163.2 (6.5)	161.3 (5.6)	164.6 (6.7)
BMI (kg/m^2^)	30.8 (5.9)	29.4 (7.4)	33.1 (4.8)	29.1 (6.1)
Ethnicity (%)				
White	83%	90%	90%	85%
African-American	17%	7%	20%	15%
Asian/Pacific Islander	0%	3%	0%	0%
Education (%)				
High school graduate	43%	50%	30%	60%
College graduate	57%	50%	70%	40%
Time since diagnosis (y)	3.6 (2.2)	3.3 (2.6)	3.5 (2.3)	3.5 (2.1)
Disease stage (%)				
*In Situ *	11%	10%	13%	10%
Stage I	54%	27%	67%	45%
Stage II	26%	47%	13%	35%
Stage IIIA	9%	17%	7%	10%
Treatment (%)				
None	6%	13%	13%	0%
Radiation only	43%	23%	47%	40%
Any chemotherapy	51%	63%	40%	60%
Hormone therapy (%)				
None	43%	30%	33%	50%
Tamoxifen	29%	23%	33%	25%
Aromatase inhibitors	29%	47%	33%	25%
Physical activity questionnaire (min per week of moderate to vigorous intensity recreational activity)	13.0 (24.0)	12.0 (20.0)	5.7 (10.2)	19.4 (29.6)

*Note*. No statistically significant differences between exercise, usual care groups, and exercise adherers versus nonadherers at baseline were observed.

**Table 2:** Percent and number of participants defined as having the metabolic syndrome at baseline and six months.

Number of metabolic syndrome criteria	Exercise baseline (*n* = 35)	Exercise 6 months (*n* = 35)	Usual care baseline (*n* = 30)	Usual care 6 months (*n* = 30)	Nonadherers baseline (*n* = 15)	Nonadherers 6 months (*n* = 15)	Adherers baseline (*n* = 20)	Adherers 6 months (*n* = 20)
0	4	7	2	3	2	6	2	1
1	5	2	7	8	5	1	0	1
2	2	5	9	6	2	5	0	0
3	10	8	6	4	3	3	7	5
4	7	11	4	7	3	5	4	6
5	7	1	2	2	5	0	2	1
Total	24	20	12	13	11	8	13	12
Percent	69%	57%	40%	43%	55%	40%	87%	80%

**Table 3:** Six-month change in metabolic syndrome variables in exercise intervention (*n* = 35) versus usual care (*n* = 30).

	Baseline (SD)	Mean change (SE)	Significance (*P* value)
Waist circumference (cm)			
Exercise	91.7 (12.0)	−1.49 (0.76)	0.508
Usual care	88.6 (15.6)	−0.75 (0.82)	
Systolic blood pressure (mm Hg)			
Exercise	123.1 (12.9)	0.66 (2.25)	0.080
Usual care	123.7 (18.7)	−5.23 (2.43)	
Diastolic blood pressure (mm Hg)			0.304
Exercise	75.2 (6.8)	0.75 (1.16)	
Usual care	76.6 (9.1)	−1.02 (1.25)	
HDL-C (mg/dL)			
Exercise	52.7 (13.2)	0.51 (1.65)	0.325
Usual care	59.1 (15.4)	−1.90 (1.78)	
Triglycerides (mg/dL)			
Exercise	123.7 (53.7)	1.40 (7.1)	0.841
Usual care	117.8 (51.6)	−0.70 (7.7)	
Glucose (mg/dL)			
Exercise	104.9 (12.7)	−1.31 (1.21)	0.029
Usual care	105.1 (10.7)	0.6 (1.3)	
Metabolic syndrome *z*-score			
Exercise	−1.7 (3.3)	−0.09 (0.39)	0.661
Usual care	−2.2 (4.2)	−0.35 (0.42)	

*Note*. Negative *z*-score indicates below cut-off. Negative mean change score indicates improvement in criterion.

**Table 4:** Six-month change in metabolic syndrome variables in exercise adherers (*n* = 20) versus nonadherers (*n* = 15).

	Baseline (SD)	Mean change (SE)	Significance (*P* value)
Waist circumference (cm)			
Adherers	89.4 (12.5)	−2.48 (1.05)	0.170
Non-adherers	94.8 (10.9)	−0.18 (1.22)	
Systolic blood pressure (mm Hg)			
Adherers	124.7 (14.4)	−1.99 (2.29)	0.091
Non-adherers	121.0 (10.6)	4.19 (2.65)	
Diastolic blood pressure (mm Hg)			
Adherers	76.2 (6.8)	−0.07 (1.00)	0.226
Non-adherers	73.9 (6.9)	1.84 (1.16)	
HDL-C (mg/dL)			
Adherers	54.8 (14.3)	2.91 (1.42)	0.016
Non-adherers	50.1 (11.5)	−2.67 (1.64)	
Triglycerides (mg/dL)			
Adherers	125.7 (63.2)	−3.13 (7.44)	0.361
Non-adherers	121.0 (39.5)	7.44 (8.60)	
Glucose (mg/dL)			
Adherers	102.6 (12.2)	−1.45 (1.58)	0.898
Non-adherers	107.9 (13.2)	−1.13 (1.83)	
Metabolic syndrome *z*-score			
Adherers	−1.92 (3.7)	−0.76 (0.37)	0.009
Non-adherers	−1.36 (2.9)	0.80 (0.42)	

WC: waist circumference, HDL-C: high-density lipoprotein cholesterol.
